# Appendicolith appendicitis: should we be operating sooner? A retrospective cohort study

**DOI:** 10.1308/rcsann.2023.0055

**Published:** 2023-08-23

**Authors:** AG Taib, A Kler, M Prayle, D Kanakalingam, M Fani, P Asaad

**Affiliations:** ^1^ Wrightington, Wigan and Leigh Teaching Hospitals NHS Foundation Trust, UK; ^2^ University of Nottingham, UK; ^3^ University of Liverpool, UK

**Keywords:** Appendectomy, Appendicitis, Humans, Retrospective studies, Tomography, X-ray computer

## Abstract

**Introduction:**

Evidence suggests that delaying an appendicectomy for up to 24 hours does not increase the risk of complicated appendicitis. Appendicoliths are a risk factor for perforation. No study has explored the temporal relationship between appendicolith presence and time to perforation. In this retrospective cohort study, we hypothesise that the presence of an appendicolith confirmed on preoperative computerised tomography scan (pCT) leads to a shorter time to complicated appendicitis.

**Methods:**

We undertook a retrospective single-centre study of patients admitted between 2018 and 2020. Inclusion criteria included: age ≥18 years, appendicitis confirmed on histopathology following an operation and a pCT scan. Complicated appendicitis was defined intraoperatively as an appendicular abscess, gangrenous or perforated appendix.

**Results:**

Some 310 patients were included in the study. Forty-five per cent presented with complicated appendicitis (*n* = 138). Appendicoliths were present in 79 (25.5%) patients. Multivariate logistic regression identified an appendicolith as a significant risk factor for perforation (odds ratio 3.50, 95% confidence interval [CI] 1.16–10.59; *p* = 0.027). Within the first 12 hours of admission, patients with an appendicolith accounted for a significantly greater proportion of those with complicated appendicitis intraoperatively compared with those without (56.7% vs 43.3%, respectively; *p* = 0.003). Within 12 hours of admission, those with an appendicolith were 2.05 times more likely to suffer from complicated appendicitis than those without (95% CI 1.28–3.29).

**Conclusions:**

Patients with an appendicolith appendicitis should be considered for an early appendicectomy. Future large-scale multicentre prospective studies are required to explore this further, perhaps informing future guidelines.

## Introduction

Acute appendicitis accounts for a significant proportion of emergency general surgery admissions.^1^ Its treatment is expedited to reduce the risk of adverse outcomes such as perforation and intraoperative complications, which are both linked to postoperative morbidity.^2^ A meta-analysis by van Dijk *et al* concluded that delaying an appendicectomy for uncomplicated appendicitis by 24 hours after admission is not associated with a greater a chance of complicated appendicitis.^3^ This is echoed by *World Journal of Emergency Surgery* (WJES) guidelines.^4^

Although faecoliths (synonym: appendicolith) are not the only suggested cause of appendicitis, the presence of an appendicolith has been shown to increase the risk of complicated appendicitis and is more likely to cause failure of treatment with antibiotics alone.^5–7^ It is postulated that a faecolith present within the appendix lumen causes stagnation of mucus and fluids produced by the mucosal epithelial cells, which enables bacterial multiplication and growth. This, along with bacterial translocation, leads to an inflammatory process. The formation of pus ensues, which increases the intraluminal pressure. When this pressure is greater than the venous outflow pressure, the appendix can no longer drain its blood, further elevating the intraluminal pressure, which now impairs arterial blood supply.^8^ This ischaemia leads to hypoxia and the loss epithelial integrity of the appendix wall, leading to gangrene and then perforation. This can cause peritonitis by releasing the bacterially enriched pus intra-abdominally, or lead to the formation of an appendicular abscess.

Since the advent of computerised tomography (CT) scans with lower radiation doses, the rate of preoperative CT (pCT) scans for appendicitis has increased,^9,10^ allowing for preoperative confirmation of an appendicolith. Because the presence of an appendicolith is associated with a higher risk of perforation, these patients should be expedited for surgery. However, no study has investigated how soon a patient with acute appendicitis due to a faecolith requires an appendicectomy.

The primary aim of this study was to explore whether the presence of an appendicolith confirmed on pCT leads to a shorter time to complicated appendicitis. The secondary aim was to confirm that an appendicolith is an independent risk factor for complicated appendicitis.

## Methods

### Ethical approval

The study was carried out in accordance with the local information data protection policy and associated codes of practice and guidelines. Ethics approval was provided for this retrospective comparison following discussion with the local research and development team.

### Inclusion and exclusion criteria

All patients who were coded for an appendicectomy on discharge using the International Classification of Diseases 10th Revision (ICD-10) at the Royal Albert Edward Infirmary, Wigan, between January 2018 and December 2020 were identified and included. A contemporaneously maintained database of 591 of these patients was accessed and reviewed retrospectively.

Patients were excluded if their age was <18 years, they were treated conservatively without an operation, if they had an elective procedure, appendicitis was not confirmed on histopathology following an operation, had incomplete records, pCT scan was not undertaken or there was no formal radiologist pCT scan report.

### Data collection

Data were collected on potential risk factors associated with a perforated appendix. These included patient demographic data. Clinical signs including guarding, peritonism and peritonitis were accessed through the electronic hospital record system. Patient vital signs on admission were also collected in a similar manner. Preoperative blood results such as white cell count (WCC) and C-reactive protein (CRP) were also included. Radiological findings such as the presence of an appendicolith, location of appendicolith, appendix dilatation and description were collected. Postoperative complications were compiled using the Clavien–Dindo classification system.^11^

### Definitions

The time of admission was defined as the time of triage in the emergency department. The time of operation was the time the appendicectomy began according to the electronic theatre management system. Time to operation was the difference between these times. The time of pCT was recorded as the time patients underwent the scan. Tachycardia, hypotension, pyrexia, tachypnoea and confusion were defined according to the National Early Warning Score 2,^12^ details of which can be found according to prior definition.^13^ Complicated appendicitis was defined as intraoperative findings of an appendicular abscess or phlegmon, gangrenous appendicitis or a perforated vermiform appendix. This definition has been used previously.^14^

### Data analysis

Using the preoperative independent variables that were significant in univariate analysis for intraoperative complicated appendicitis, a multivariate logistic regression model was used. To test for the significance of continuous variables during univariate analysis, an independent samples *t*-test and Mann–Whitney *U* test or Kruskal–Wallis test were used for parametric and non-parametric data, respectively. A chi-squared test of independence was performed to examine the significance of categorical variables for univariate analysis and the relationship between the presence of an appendicolith and time to complicated appendicitis. Values of *p* < 0.05 were considered statistically significant. Statistical analysis was performed using IBM^®^ SPSS^®^ Statistics v.28.0 (Armonk, NY, USA).

## Results

### Univariate analysis

#### Demographic data

After fulfilling the inclusion and exclusion criteria a total of 310 patients were included in the study: 171 patients had uncomplicated appendicitis and 138 had complicated appendicitis. Patient demographic data and time to intervention information are illustrated in Table 1. The median age of patients in the complicated appendicitis group was significantly greater than in the uncomplicated group (47.5 years, interquartile range [IQR] 29.3 years vs 35 years, IQR 23.0 respectively; *p* < 0.001). Almost half the patients included in the study were female (47.9%, *n* = 148), which was similar between both cohorts (*p* = 0.064).

**Table 1:** Patient demographic data and time to intervention information

Characteristics	n*	All patients	Uncomplicated appendicitis	Complicated appendicitis	*p-*value
Demographic					
Age, median (IQR), years	309	39.5 (26.0)	35.0 (23.0)	47.5 (29.3)	**<0.001**
Sex ratio, male : female	309	161:148	81:90	80:58	0.064
BMI, mean (sd), kg/m^2^	137	28.5 (6.4)	29.2 (7.2)	27.6 (5.3)	0.144
Timing, median (IQR), hours	309				
Time from admission to operation		21.4 (13.8)	22.7 (15.8)	18.7 (13.05)	**0.006**
Time from admission to CT scan		7.45 (9.3)	8.5 (10.7)	6.7 (7.7)	0.075
Time from CT scan to operation		10.8 (13.4)	11.5 (14.1)	10.0 (12.6)	0.134

*n < 310, patients excluded because of missing data

BMI = body mass index; CT = computer tomography; IQR = interquartile range; sd = standard deviation

The median time from admission to operation was shorter by 4 hours in those with complicated appendicitis compared with patients with uncomplicated appendicitis (*p* = 0.006). The median time from pCT to theatre was 10.8 hours (IQR 13.4), which was similar between both cohorts (*p* = 0.134).

### Preoperative clinical, laboratory and radiological findings

Those with an intraoperative finding of complicated appendicitis had a significantly longer duration of abdominal pain compared with those with uncomplicated appendicitis (*p* < 0.001). No significant difference was found between the patient-reported length of abdominal pain and time to theatre (*p* = 0.940). This suggested that the duration of abdominal pain did not influence time from admission to operation. Table 2 also highlights more advanced clinical (guarding or peritonism, tachycardia, pyrexia) and laboratory findings (WCC and CRP) in complicated appendicitis patients which were significant.

**Table 2:** Clinical, laboratory and radiological findings

Characteristics	n*	All patients	Uncomplicated appendicitis	Complicated appendicitis	*p-*value
Clinical, *n* (%)					
Duration of abdominal pain on admission, hours	308				**<0.001**
≤12		47 (15.3)	37 (21.6)	10 (7.4)	
>12 to ≤24		105 (34.1)	67 (39.2)	38 (27.9)	
>24 to ≤48		156 (50.6)	67 (39.2)	88 (64.7)	
Clinical examination findings	309				**0.003**
No guarding		180 (58.3)	110 (64.3)	70 (50.7)	
Local guarding or peritonism		118 (38.2)	52 (30.4)	66 (47.8)	
Peritonitis		11 (3.6)	9 (5.3)	2 (1.4)	
Tachycardia	309	83 (26.9)	38 (22.2)	45 (32.6)	**0.041**
Hypotension	309	5 (1.6)	4 (2.3)	1 (0.7)	0.263
Pyrexia	309	47 (15.2)	15 (8.8)	32 (23.2)	**<0.001**
Tachypnoea	309	26 (8.4)	15 (8.8)	11 (8.0)	0.801
Confusion	309	2 (0.6)	1 (0.6)	1 (0.7)	0.879
Preoperative bloods, median (IQR)					
WCC, 10^9^/l	309	13.2	13.0 (6.1)	14.0 (5.8)	**0.007**
CRP, mg/l	310	55	29 (140)	125 (69)	**<0.001**
Radiological findings					
CT description, *n* (%)	309				**<0.001**
Appendicitis		246 (79.6)	165 (96.5)	81 (58.7)	
Abscess or phlegmon		18 (5.8)	3 (1.8)	15 (10.9)	
Perforation		43 (13.9)	3 (1.8)	40 (29.0)	
Other		2 (0.6)	0 (0.0)	2 (1.4)	
Appendicolith present, *n* (%)	310	79 (25.5)	24 (14.0)	55 (39.9)	**<0.001**
Appendix dilatation, median (IQR), mm	151	11.0 (4.0)	10 (3)	12 (5)	**<0.001**
Location of appendicolith, *n* (%)	74				0.931
Proximal		47 (63.5)	14(66.7)	33 (62.3)	
Mid		11 (14.9)	3 (14.3)	8 (15.1)	
Distal		16 (21.6)	4 (19.0)	12 (22.6)	

*n < 310, patients excluded because of missing data

CRP = C-reactive protein; CT = computer tomography scan; IQR = interquartile range; WCC = white cell count

Only 14% (*n* = 10) of patients with uncomplicated appendicitis intraoperatively had a pCT that suggested the presence of an appendicolith (Table 2). However, of those with an intraoperative finding of complicated appendicitis, 39.9% (*n* = 55) exhibited an appendicolith on pCT (*p* < 0.001). The appendix was also more dilated in the complicated cohort than in the uncomplicated cohort (median 12mm, IQR 5 vs 10mm, IQR 3mm respectively; *p* < 0.001).

### Operative findings

Most patients had a radiological diagnosis of uncomplicated appendicitis on admission (79.6%, *n* = 246; Table 2). However, at operation, the proportion of patients with uncomplicated appendicitis decreased to 55.3% (*n* = 171; Table 3). Of those patients with an intraoperative finding of complicated appendicitis the majority were identified as having a perforation (44.9%, *n* = 62) or necrosis or gangrene of the appendix (43.5%, *n* = 60). Sixteen patients (11.6%) had an intraoperative finding of an abscess or phlegmon. Other intraoperative findings are described in Table 3.

**Table 3:** Intraoperative findings and postoperative characteristics

Characteristics	n*	All patients	Uncomplicated appendicitis	Complicated appendicitis	*p-*value
Operative findings, *n* (%)					
Intraoperative findings	309				
Uncomplicated appendicitis		171 (55.3)	171 (100.0)	0 (0.0)	
Necrosis or gangrene		60 (19.4)	0 (0.0)	60 (43.5)	
Abscess or phlegmon		16 (5.2)	0 (0.0)	16 (11.6)	
Peroration		62 (20.1)	0 (0.0)	62 (44.9)	
Intraoperative peritoneal soiling	309				**<0.001**
None or serous fluid		204 (66.0)	150 (87.7)	54 (39.1)	
Turbid fluid		18 (5.8)	8 (4.7)	10 (7.2)	
Pus		83 (26.9)	13 (7.6)	70 (50.7)	
Bowel content		4 (1.3)	0 (0.0)	4 (2.9)	
Postoperative					
Length of stay, median (IQR), days	308	2 (2)	1 (0)	2 (3)	**<0.001**
Postoperative complications	309				**0.002**
No complications, *n* (%)		263 (85.1)	158 (92.4)	105 (76.1)	
Clavien–Dindo I		5 (1.6)	2 (1.2)	3 (2.2)	
Clavien–Dindo II		26 (8.4)	7 (4.1)	19 (13.8)	
Clavien–Dindo III		15 (4.9)	4 (2.3)	11 (7.9)	

*n < 310, patients excluded because of missing data

IQR = interquartile range

### Postoperative findings

Patients who had uncomplicated appendicitis had a shorter median length of hospital stay than those with complicated appendicitis (1 day, IQR 0 vs 2 days, IQR 3 days respectively; *p* < 0.001). Furthermore, postoperative complications were also less frequent and severe in the uncomplicated cohort (*p* = 0.002) as highlighted in Table 3.

### Multivariate logistic regression model to predict complicated appendicitis

Multivariate logistic regression was performed to assess the impact of significant independent variables in the univariate analysis on the likelihood of a patient suffering from complicated appendicitis. Ten variables were included in the model (Table 4). Six of the independent variables made a unique statistically significant contribution to the model.

**Table 4:** Multivariate logistic regression model used to predict complicated appendicitis using significant variables from univariate analysis

Predictor	Odds ratio	95% CI for odds ratio		
		Lower	Upper	Significance
Age at presentation (years)	1.04	1.01	1.08	0.010*
Duration of abdominal pain				0.500
Duration of abdominal pain ≤24 hours	1.42	0.31	6.44	0.646
Duration of abdominal pain ≤48 hours	2.30	0.49	10.83	0.291
Examination signs				0.005*
Localised guarding	3.57	1.37	9.31	0.009*
Peritonitis	0.17	0.02	1.94	0.155
Tachycardia present	1.72	0.54	5.45	0.355
Pyrexia present	3.70	1.03	13.31	0.046*
Preoperative C-reactive protein (mg/l)	1.01	1.00†	1.02	0.013*
Preoperative white cell count (10^9^/l)	1.12	0.99	1.28	0.069
Time from admission to operation (hours)	1.00	0.96	1.04	0.928
Appendicolith present on preoperative CT	3.50	1.16	10.59	0.027*
Appendix dilatation (mm)	1.25	1.06	1.47	0.008*

*Denotes significant predictors of complicated appendicitis in the regression model

†Lower 95% CI for C-reactive protein is 1.002 therefore because >1 is a significant predictor

CI = confidence interval; CT = computed tomography

The strongest predictor of complicated appendicitis at operation was admission pyrexia, recording an odds ratio (OR) of 3.70 (95% confidence interval [CI] 1.03–13.31; *p* = 0.046). An appendicolith on pCT scan was associated with a 3.5 times greater chance of complicated appendicitis (OR 3.50, 95% CI 1.16–10.59; *p* = 0.027). Increased appendix dilatation was also associated with a greater risk complicated appendicitis in the model (OR 1.25, 95% CI 1.06–1.47; *p* = 0.008).

### Temporal relationship between appendicolith and complicated appendicitis

**Figure 1:**
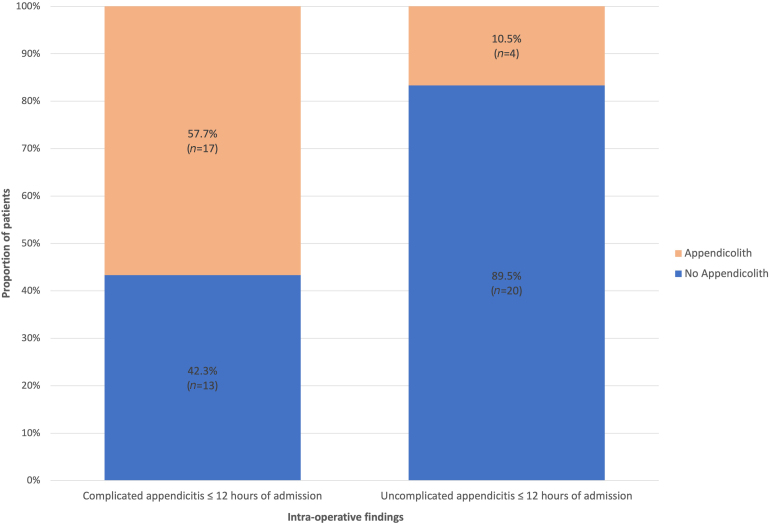
Bar chart comparing the proportion of patients with uncomplicated and complicated appendicitis within 12 hours of admission and the prevalence of appendicoliths on preoperative computed tomography scan

Within the first 12 hours of admission, those with an appendicolith accounted for a significantly greater proportion of patients with complicated appendicitis intraoperatively (appendicolith: 56.7%, *n* = 17 vs no appendicolith: 43.3%, *n* = 13; *p* = 0.003). In those with uncomplicated appendicitis at ≤12 hours from admission, the majority did not have an appendicolith on pCT (no appendicolith: 83.3%, *n* = 20 vs appendicolith: 16.7%, *n* = 4; *p* = 0.003). This is illustrated in Figure 1. Within 12 hours those with an appendicolith were 2.05 times more likely to suffer from complicated appendicitis than those without (95% CI 1.28–3.29; *p* = 0.003).

## Discussion

This retrospective cohort study demonstrated that the presence of an appendicolith on pCT is associated with a shorter time to complicated appendicitis. It suggests that those with an appendicolith are twice as likely to suffer complicated appendicitis within 12 hours of admission compared with patients with acute appendicitis without an appendicolith (relative risk 2.05, 95% CI 1.28–3.29; *p* = 0.003). Furthermore, it also confirmed that the presence of an appendicolith is an independent risk factor for complicated appendicitis using a multivariate logistic regression model (OR 3.50, 95% CI 1.16–10.59; *p* = 0.027).

In this study of 310 patients, approximately one in four were reported to have an appendicolith on pCT, which is similar to a previously reported prevalence of 27% during preoperative imaging in a randomised trial comparing antibiotics to surgery, in 1,552 patients.^7^ The prevalence of an appendicolith in complicated appendicitis and uncomplicated appendicitis in this study was 39.9% and 14.0%, respectively, which are the same proportions reported in a retrospective study.^15^ However, Singh *et al*^15^ defined complicated appendicitis as perforation only. The median time to theatre in this study was 21.4 hours, which is in keeping with WJES guidelines;^4^ however, those with complicated appendicitis diagnosed intraoperatively had a significantly shorter time to theatre compared with those without. This is expected because patients with complicated appendicitis are expected to be clinically unwell, prompting a quicker time to theatre.

Our study suggests an appendicolith on pCT is strongly associated with negative outcomes (OR 3.50, 95% CI 1.16–10.59; *p* = 0.027). However, it is not routinely used to expedite the management of acute appendicitis in patients. Pyrexia and guarding on examination are typically used to triage and prioritise patients for theatre.^16–19^ In our regression model, these findings had predictive values for complicated appendicitis comparable with the presence of an appendicolith. With novel evidence from this study regarding time to perforation echoed by previous research confirming that an appendicolith is related to negative outcomes in acute appendicitis,^7,18,20^ and the increased use of pCT for the diagnosis of appendicitis,^9,21^ we suggest that acute appendicolith appendicitis should be considered for an early operation akin to the patient suffering from pyrexia or guarding.

Although the presence of an appendicolith is linked to complicated appendicitis, controversy surrounds the hypothesis that it is a direct cause of appendicitis. In several studies, faecoliths are present within the lumen of a substantial portion of normal appendix specimens or in diagnostic imaging, suggesting they are incidental findings only.^15,22–24^ However, Mällinen *et al* compared histopathological differences between acute appendicitis with and without an appendicolith. This highlighted that those with an appendicolith showed greater mucosal ulcerations, micro-abscesses and a different T-helper cell immune response. Mällinen *et al* suggest that acute appendicolith appendicitis should be treated as an emergent finding and is a different entity to acute appendicitis alone,^6^ which may be due to an alternate cause altogether.

There is also debate surrounding the in-hospital delay of an appendicectomy. A large multicentre retrospective in study in Washington State suggests that in-hospital time prior to surgery is not associated with a higher rate of perforation in acute appendicitis. The authors suggest that perforation is a prehospital occurrence that is not time related. However, it is worth noting in this study that mean time to theatre was 8.6 hours.^16^ In our study, based in the Nation Health Service, the median time to theatre was 21.4 hours. Therefore, in-hospital delay in this context is not comparable. Furthermore, in our study, approximately 20% of patients on admission had a radiological diagnosis of complicated appendicitis. At operation, this increased to 45%. Although controversy exists regarding the sensitivity of pCT in detecting perforated appendicitis,^25^ it suggests that complicated appendicitis may not solely be a prehospital occurrence. The WJES guidelines on the timing of an appendicectomy for acute uncomplicated appendicitis include two meta-analyses, both suggesting that delaying an appendicectomy for up to 24 hours is acceptable.^3,4,14^ However, predicting the course of uncomplicated appendicitis to complicated appendicitis within 24 hours is difficult.

Appendicitis is a clinical diagnosis; however, patients may undergo pCT scan to assist the clinician in deciding on the operative or nonoperative management of appendicitis.^26,27^ pCT is also used if there is sufficient concern from the clinician for the presence of an appendiceal neoplasm, or if peritonitis or sepsis are present but the aetiology unclear.^26^ Therefore, it could be argued that patients in this study who underwent a pCT were more likely to suffer from adverse outcomes. Conversely, 171 patients in this study had uncomplicated appendicitis intraoperatively, suggesting otherwise. It does, however, imply that the use preoperative imaging for the diagnosis of appendicitis is increasing.^9,21^ The reported sensitivity and specificity of modern pCT imaging in detecting an appendicolith is 66% and 82%, respectively.^28^ This may be falsely low because appendicoliths seen on pCT may be removed from histopathology specimens by handling of the appendix intraoperatively.^6^ With significant evidence suggesting that an appendicolith appendicitis is a risk factor for failure of nonoperative management,^7,29^ along with the increasing trend of preoperative diagnostic imaging providing additional radiological data to our clinical practice,^9,21^ we advocate that the presence of an appendicolith should be considered in the management of acute appendicitis patients despite its low sensitivity when pCT is utilised.

Strengths of the study include the inclusion of patients with confirmed appendicitis on histopathology only. This provides unique information on the progression of acute appendicitis with and without an appendicolith. Although this resulted in the exclusion of patients who were managed nonoperatively for their acute appendicolith appendicitis, there is evidence in the literature regarding this,^7,29,30^ and the use of an intraoperative diagnosis of complicated appendicitis strengthened the study by not relying on pCT, which may be undertaken before time to complicated appendicitis or be inaccurate.^31^ To account for inter-surgeon variability, future studies could use a validated grading system to score the intraoperative severity of acute appendicitis. Limitations of the study include its retrospective nature and the risk of confounding variables. However, the use of a multivariate logistic regression model accounted for this. Its single-centre nature, part of which extended into the COVID-19 pandemic, may also limit its generalisability to current practice and other centres. During this time, both nationally and locally, there was an increase in the use of pCT to confirm diagnosis to assist decisions regarding conservative management of appendicitis.^9,27^

## Conclusion

This novel study suggests that patients presenting with acute appendicitis, with an appendicolith observed on preoperative diagnostic imaging, should be considered for an early appendicectomy owing to a greater risk of complicated appendicitis within 12 hours. This study highlights that complicated appendicitis is associated with greater and more serious postoperative complications, along with a greater length of hospital stay. Therefore, this should be avoided. Future large-scale multicentre prospective studies are required to explore this further, perhaps informing future guidelines.
